# Zika Virus Trafficking and Interactions in the Human Male Reproductive Tract

**DOI:** 10.3390/pathogens7020051

**Published:** 2018-05-11

**Authors:** Lucia Regina Cangussu da Silva

**Affiliations:** Departamento de Ciências da Saúde, Universidade Salgado de Oliveira, Juiz de Fora, Minas Gerais, CEP 36036-000, Brazil; prof.luciacangussu@gmail.com; Tel.: +55-32-98854-9748

**Keywords:** Zika virus, sexual transmission, male reproductive tract, human reproduction

## Abstract

Sexual transmission of Zika virus (ZIKV) is a matter of great concern. Infectious viral particles can be shed in semen for as long as six months after infection and can be transferred to male and female sexual partners during unprotected sexual intercourse. The virus can be found inside spermatozoa and could be directly transferred to the oocyte during fertilization. Sexual transmission of ZIKV can contribute to the rise in number of infected individuals in endemic areas as well as in countries where the mosquito vector does not thrive. There is also the possibility, as has been demonstrated in mouse models, that the vaginal deposition of ZIKV particles present in semen could lead to congenital syndrome. In this paper, we review the current literature to understand ZIKV trafficking from the bloodstream to the human male reproductive tract and viral interactions with host cells in interstitial spaces, tubule walls, annexed glands and semen. We hope to highlight gaps to be filled by future research and potential routes for vaccine and antiviral development.

## 1. Introduction

The male reproductive tract (MRT) has become a site of great interest in virology since the emergence of HIV infections in the 80s [1]. At least 27 viruses that cause viremia can be found in human semen [2]. They belong to very diverse families and may cause diseases of variable symptoms and outcomes [3]. Viruses found in semen have either crossed the endothelial lining of vessels during viremia, have exfoliated from the penile epithelia during sample collection by the patient or have been acquired during sexual intercourse from an infected partner.

Viruses do not necessarily replicate in the MRT, but can be maintained inside cells or as free particles in secretions for years [4]. The site of viral replication in the MRT is usually unknown for most viruses [5], inciting further research to elucidate host–virus interactions. Viral infection of the MRT may result in infertility, greater susceptibility to sexually transmitted diseases, integration of viral genome into host cells, cancer, immune deficiency, changes in hormone levels, orchitis, epididymitis, burning sensation in the urethra, persistence of viral replication and shedding and long-term transmission to sexual partners [6,7,8].

The dynamics of viral infection in the MRT are extremely variable depending on host and viral factors such as the ability of the virus to cross the blood–testis barrier, infect cells in the interstitial space and the wall of seminiferous and epididymal tubules, escape or manipulate innate immune responses, especially inflammation, viral latency mechanisms, host cell susceptibility and permissiveness to viral infection, among others [7,8].

The Ebola outbreak in Africa in 2014 raised further concern about the long-term persistence and transmission of viruses through semen. Using comparative genomics, it was proven that Ebola virus could be transmitted through sexual intercourse from male-to-female up to 155 days after the male first blood sample tested positive for Ebola [9]. Viral shedding in semen persisted for at least 199 days (175 days after Ebola clearance from the patient’s blood). Ebolavirus RNA has been detected in semen up to 18 months after patient discharge from an Ebola treatment unit [10]. Therefore, the MRT is an important reservoir for Ebola virus and sexual transmission can contribute to the upsurge of cases in Ebola endemic areas if proper prevention strategies are not adopted.

The 2015 Zika virus (ZIKV) outbreak in Brazil and the subsequent demonstration of the association between ZIKV and microcephaly [11,12] raised the concern about the presence of this arbovirus in semen and the possibility of sexual transmission [13], which would have a great impact in public health in the country [14,15]. Moreover, sexual transmission could be a plausible explanation for the surprisingly high number of suspected new cases of microcephaly reported to the Ministry of Health in Brazil at that time. There was an urge among scientists to test semen and vaginal secretions for the presence of ZIKV RNA and to determine if ZIKV particles shed in these body fluids were infectious, especially after two cases of possible sexual transmission of the infection were reported in the USA [16,17]. Since then, ZIKV has been detected in semen [18,19] and in vaginal secretions [20,21] and the virus has been found to persist in the MRT favoring transmission through unprotected sexual intercourse [20,22,23].

The interest in viral shedding in semen continued to grow as the Brazilian outbreak continued. In October 2016, the first report of prolonged shedding of Chikungunya RNA in semen was published by a group of Brazilian researchers working in the city of Salvador, Bahia [5]. The viral RNA was detected in semen samples from a 25-year-old man up to 30 days after symptom onset.

Brazil has been facing the risk of yellow fever urbanization, and as the number of cases rise in large urban centers, such as the city of São Paulo, the risk of acquiring the disease through the bytes of infected *Aedes (Stegomyia) aegypti* and *Aedes albopictus* also increases in these areas [24]. Equally important is the fact that the yellow fever virus (YFV) RNA has recently been detected in a semen sample of a Brazilian patient during the recovery phase [25]. Although viral infectivity in semen was not proven, the authors could culture the virus from a urine sample collected in the same date as the semen sample. Further studies are needed to precisely determine the dynamics of YFV shedding in semen.

It becomes clear from this brief introduction that the MRT is an appropriate site for replication and persistence of several viruses, including arboviruses that are endemic in tropical countries posing a serious threat to populations in which unprotected sexual intercourses occur. The aim of the present review is to address the trafficking of ZIKV from the human blood to the MRT and its interactions with cells, secretions and immune responses, and to highlight the gaps in our current understanding of ZIKV interactions within the human MRT. We hope that a better understanding of ZIKV infections in males will contribute to the definition of new research goals, better reproductive recommendations and new strategies for vaccine development in a Zika threatened world.

## 2. A Brief Historical Perspective of ZIKV Sexual Transmission

One of the first pieces of evidence that ZIKV could be sexually transmitted came from a case report published in 2011 (Table 1) in which a woman developed arthralgia and exanthema after having unprotected vaginal intercourse with her husband, who had developed arboviral infection symptoms and prostatitis with mild dysuria and perineal pain followed by hematospermia after returning from Senegal [16]. Additionally, two cases of probable sexual transmission that occurred in May 2014 were retrospectively diagnosed in Italy and published early in 2016 [26].

In February 2015, Musso and his collaborators [27] detected the presence of infectious ZIKV particles in two samples of semen from a patient who had arboviral infection symptoms twice in a 10 week period. He sought medical care when he developed hematospermia. ZIKV load in semen reached 2.9 × 10^7^ copies/mL at ten weeks after symptom onset and declined to 1.1 × 10^7^ copies/mL after three days. ZIKV particles could be cultured in Vero cells proving their viability and potential for sexual transmission. A viral load as high as 400 million copies/mL of semen was reported in March 2016. These particles also replicated in cell culture adding further evidence that ZIKV could be sexually transmitted [28]. In May, another report confirmed the presence of this virus in semen [29].

The association between ZIKV and microcephaly was already suspected [34] and sexual transmission of this virus could have serious consequences for the Brazilian health system, especially if one considered that 80% of the infected population were probably asymptomatic [35]. It has been reported that ZIKV RNA can be detected in semen samples of asymptomatic blood donors revealing that there is a risk for sexual transmission even when the individual is asymptomatic [30].

In April 2016, the first report of male-to-male transmission of ZIKV between mutually monogamous men was published [31]. One of the patients had recently travelled to a country with confirmed autochthonous transmission of ZIKV and his partner had not left Dallas, Texas, USA. They engaged in condomless anal sex one day before and one day after symptom onset on the index case. Seven days later, the partner developed fever, myalgia, and other arboviral infection signs and symptoms. Plaque-reduction neutralization tests indicated that he had been infected with ZIKV.

The first report of ZIKV RNA detection in cervical mucus was published in July 2016 [32]. The female patient declared the use of condom in all her sexual intercourses, so sexual transmission was ruled out. Although the infectivity of the particles was not determined, it raised the concern that women could harbor the virus in their genital tract for prolonged periods [36,37] and serve as viral reservoirs for subsequent transfer of ZIKV to men during unprotected sexual intercourse. Not surprisingly, sexual transmission of ZIKV from a female to a male was reported that same month. The female had travelled to an area of ongoing ZIKV transmission and she had a condomless vaginal intercourse with a male partner after arrival in New York city. She developed typical symptoms of arboviral infection the next day and her partner six days later. ZIKV RNA was detected in serum and urine samples from both individuals [33]. Interestingly, the male had not travelled outside the USA, did not report having sex with other partners and local ZIKV transmission had not been reported in the city.

The abovementioned reports provide solid evidence that supports ZIKV transmission through oral, vaginal and anal intercourses [38] from symptomatic or asymptomatic partners. The virus has been shown to persist in the MRT and female reproductive tract (FRT) and to be shed and viable in semen and in vaginal secretions for a long period [37,39] favoring sexual transmission.

Many countries have now reported cases of sexually transmitted ZIKV infections [17,22,38,40,41,42]. Most of these cases involve a sexual partner who travelled to countries where the virus is circulating [43,44,45]. This mode of transmission makes ZIKV infection of great concern even in countries where the mosquito vector is not found. The development of guidelines for safe sexual intercourses is crucial to avoid the transmission of the disease and to reduce the frequency of unintended pregnancies associated with the risk of neurological lesions in fetuses and newborns. Counseling for couples planning a pregnancy is also important [46,47], not only in countries where the virus circulates, but also for travelers returning from these areas. Unfortunately, despite all these efforts, it is possible that ZIKV sexual transmission will continue to be a matter of great concern for public health in developed and underdeveloped countries for quite some time.

## 3. Interactions of ZIKV in the Male Reproductive Tract

**Figure 1:**
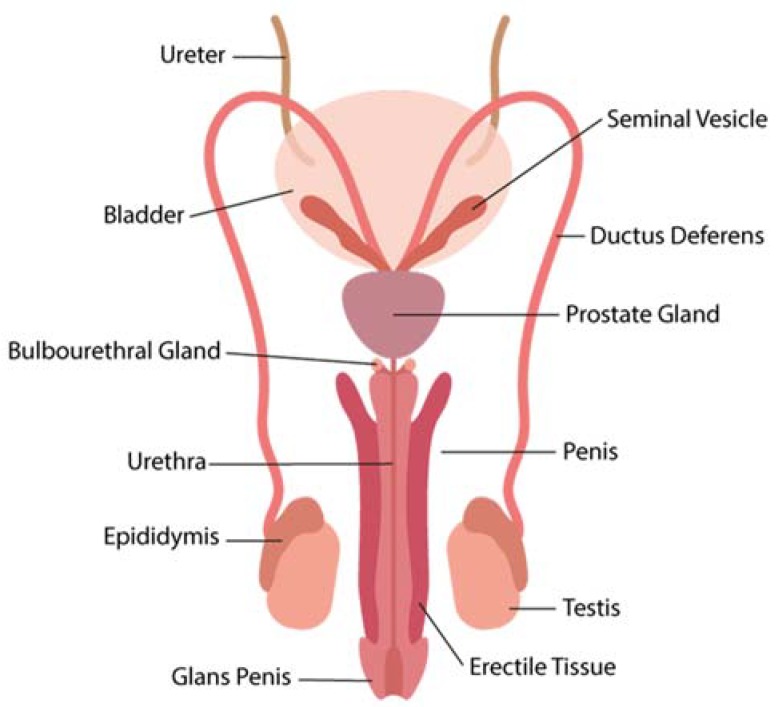
Schematic representation of the male reproductive tract.

The MRT is comprised of the testis, epididymis, vas deferens, prostate, seminal vesicle, bulbourethral glands and the penile urethra (Figure 1). Two main functions of the MRT are (1) the production, maturation and protection of spermatozoa from immune aggression and infectious agents and (2) the production of hormones. To achieve these goals, the MRT has evolved a series of structural and functional features which are far beyond the scope of the present review. For those seeking for a deeper understanding of these features, we recommend reading comprehensive reviews found in the literature [48,49,50].

To favor human reproduction, the MRT must reduce the chances of contact of neo-antigens present on spermatogonial stem cells, spermatocytes and spermatozoa with immune cells, to prevent the stimulation of auto-immune responses. Additionally, it must establish a certain degree of immune suppression and tolerance to protect these cells against immune attack while keeping the ability to fight intruding infectious agents, including viruses [51].

The testis may serve as a reservoir for viruses after their hematogenous dissemination [3] and even after they have been cleared from the blood. This persistence has been clearly demonstrated for HIV in humans [52]. Persistence in the testis requires that viruses cross the endothelial layer, reach the interstitial space, infect or ideally replicate in cells present in this location or in the seminiferous tubule wall, and evade or modulate immune responses. Viruses can alter the blood testicular barrier (BTB) making it leakier or infect Sertoli cells to reach the adluminal face of the seminiferous tubules either free or inside the motile spermatozoa. Thus, viruses must find a portal of entry into the testis, invade cells and modulate immune responses to favor their replication or persistence in the MRT.

The detection of ZIKV RNA in semen was a clear demonstration that this virus could reach the MRT, but the exact mechanisms of invasion, replication and persistence were unknown at that time [27]. Understanding how ZIKV passes from the blood into the MRT and its interactions with different cells types might help in the development of vaccines and antiviral drugs that would prevent sexual transmission and the deleterious effects in pregnancy [53]. Moreover, ZIKV poses a risk to semen banks and to human fertilization strategies as it can be shed persistently for months in semen of men who are infected by the virus [39,54]. An aggravating situation in this context is that sperm preparation procedures that reduce the risk of HIV and HCV transmission are not effective against ZIKV [55].

Several mechanisms can lead to altered or impaired sperm production, subfertility or infertility because of viral infections in the MRT. Reduced generation of germ cells, germ cell apoptosis, sperm cell abnormalities, low sperm cell generation, altered sperm cell capacitation, inflammation resulting in immune cell infiltration, activation of autoimmune responses, accessory glands infection and duct obstruction have been reported [56]. Unfortunately, most of the studies on ZIKV biology in the MRT have been carried out in animal models, so care must be taken not to assume that the same interactions and outcomes translate to the human MRT. We will attempt to focus on studies carried on human patients and on human cell cultures in vitro to have a clearer picture of the potential effects of this virus on male reproductive health.

### 3.1. Zika Virus Interactions in the Testis

#### 3.1.1. Zika Virus and the Hematotesticular Barrier

**Figure 2:**
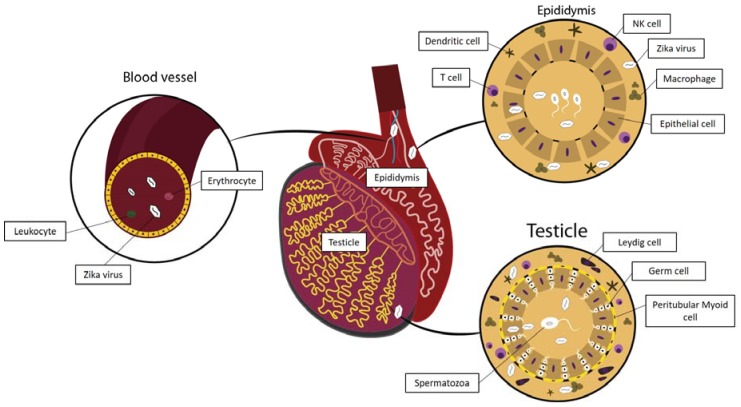
Probable Zika virus trafficking and interactions within the male reproductive tract.

One intriguing characteristic of ZIKV infection is the long time required for viral clearance from the bloodstream [57,58]. Detection of ZIKV RNA in blood samples for up to 120 days was reported in males in a cohort study [55]. The persistence is longer in the cell fraction than in plasma [59,60], suggesting that the virus can evade innate and adaptive antiviral immune responses favoring its intracellular persistence. ZIKV RNA is detected up to 81 days in erythrocytes in the presence of a robust humoral response with neutralizing antibodies against the virus [37], and for approximately 150 days in white blood cells, the latter exhibiting a higher viral load than platelets [59]. Similar results have been reported for other flaviviruses such as West Nile virus and Dengue virus [61,62,63,64]. These findings not only point to the potential for blood transfusion transmission of ZIKV, which was suspected early in the outbreak in French Polynesia [65] and in Brazil [66], but also shows that ZIKV has plenty of time to cross blood–tissue barriers in the male body reaching immune privileged sites such as the testis and establishing a reservoir for persistent infection (Figure 2).

On reaching the testicles, circulating ZIKV particles would need to transmigrate through the endothelium lining of the capillary network to reach the interstitial space. There is no clear demonstration that the virus infects endothelial cells in the testicular vasculature (Figure 2), but ZIKV has been shown to infect endothelial cells from many tissues, including from aortic and coronary artery where productive infection is established [67,68,69]. In human brain microvascular endothelial cells, ZIKV infection activates genes involved in viral persistence, cell survival, inhibition of apoptosis, and the production of chemokines, among others [70]. ZIKV evades the interferon-α (IFN-α) antiviral effect, induces host cell survival mechanisms, replicates and spreads in monolayers, even when the virus is inoculated in low numbers. These endothelial cells remain 100% viable and highly productive of viral particles. Even so, they retain their capacity to control the paracellular flow of molecules. Exactly the opposite is seen in endothelial cells infected with Dengue virus [71,72], which may cause hemorrhagic syndromes not commonly seen in ZIKV infected patients.

ZIKV particles are released to the adluminal and luminal surfaces of the monolayers of human brain microvascular endothelial cells proving that, at the end of the replication cycle, the virus can be delivered to the brain, an immune privileged site. It is possible that a similar process could take place in the testis. ZIKV particles persisting in the male blood would adsorb to and enter the apical surface of the endothelial cells lining the capillaries in the testis, replicate in their cytoplasm and be released in the basolateral membrane that faces Leydig cells, macrophages, dendritic cells and fibroblast in the interstitial space.

Some evidence suggests that this might be the case. It has been demonstrated that endothelial cells have several receptors for flaviviruses, including AXL, a receptor from the tyrosine kinase family which is a co-receptor and entry facilitator for ZIKV in humans [69,73,74]. Signaling through this receptor is known to suppress innate immune responses and favor viral replication [75]. Hamel and co-workers [76] were the first to identify ZIKV innate immune receptors in human dermal fibroblasts (TLR3), immature dendritic cells (DC-SIGN) and keratinocytes (AXL). Additionally, endothelial cells are capable of sensing viral RNA through RIG-I and plasmacytoid DCs through TLR7 resulting in the production of pro-inflammatory cytokines [77]. If present in interstitial cells in the testis, they could facilitate ZIKV infection. In support of this hypothesis, epithelial cells and fibroblasts from different species and tissues, including vascular endothelial cells, were permissive to ZIKV and able to replicate it to high titers [78]. The fact that chloroquine inhibits ZIKV infection of these cells in vitro [79] suggests that this drug could help control viral replication in blood–tissue barriers.

#### 3.1.2. Zika Virus Interactions with Cells Present in the Testicular Interstitial Spaces

In the interstitial spaces between seminiferous tubules one can find a network of capillaries surrounded by connective tissue containing immune cells and the testosterone producing Leydig cells [80]. The cells present in the interstitial spaces can interact with viruses that invade the male reproductive tract [7].

Leydig cells (Figure 2) have not been proven to be infected by ZIKV in humans, but a trend to low testosterone concentrations and significantly higher luteinizing hormone which are markers of testicular failure have been reported [55]. Leydig cells are considered the first line of defense for viruses arriving in the testicular environment [81] from the blood stream and are targeted by Mumps virus and Cytomegalovirus causing orchitis [7]. They seem to be infected later than Sertoli cells in mouse models used to study ZIKV infection and should be studied in greater detail in humans [82].

Testicular macrophages (Figure 2) are also part of the first line of defense in the peritubular compartment of the seminiferous tubules [83]. ZIKV infects human testicular macrophages in vitro, but the replication is not as vigorous as in human Sertoli cells and peaks 24 h after inoculation. Infected macrophages show a moderate increase in the production of IFN-α and of interferon induced protein with tetratricopeptide repeats 1(IFIT-1), both involved in the antiviral activity against several RNA viruses [84]. Similar results have been described for ZIKV infection in human lung epithelial cells [85], where the rise in transcriptional activity of IFN and IFIT genes was interpreted as a viral strategy to delay host cell apoptosis and favor viral initial replication.

ZIKV infected macrophages secrete pro-inflammatory cytokines [84] including TNF- α, IL-1 α, IL-8 and chemokines such as GRO (chemoattractant for neutrophils), IP-10 (Interferon induced chemokine leading to the stimulation of monocytes, NK and T-cell migration and to the modulation of adhesion molecule expression) and MCP-1 (monocyte chemoattractant protein 1). Similar results have been reported for ZIKV infected Hofbauer cells [86], which are fetal macrophages found in the placental villi. These cytokines and chemokines produced by infected macrophages led to the degradation of ZO-1, a protein present in the tight junction between adjacent Sertoli cells, increasing the permeability of the BTB. Altogether, these results show that ZIKV infection of testicular human macrophages increases the inflammatory status in the peritubular region of the seminiferous tubule, contributes to leukocyte migration and to the disruption of the Sertoli cell barrier function. These macrophage-induced alterations of the BTB can facilitate ZIKV infection of germ cells, Sertoli cells, spermatids and spermatozoa and also allow the virus to reach the lumen of the seminiferous tubule where it can be carried as a free virion to the epididymis, urethra and to the FRT. Macrophages in the human MRT warrant further investigation not only to determine their origin, but also their precise phenotype and the dual role they play in fighting ZIKV invasion and in inducing damage to the seminiferous tubules wall and possibly, spermatogenesis.

Zika virus infects monocyte derived human Dendritic cells (DCs) (Figure 2) in vitro, but considerable variation is seen in viral replication in DCs from different individuals and in the replication kinetics between viral strains [87]. The historical African lineages replicate more rapidly than the Asian lineage and are the only ones to induce DC death. The Asian lineage used in this study was very similar to the one that circulated in Brazil in 2015 and its replication in DCs peaked 12 h post-infection and plateaued between 48 h and 72 h. As viral particles were produced, the number of infected DCs also raised proving that the viruses produced by these DCs were infectious to the cells present in the culture. The fact that the Asian strain did not induce DC cell death indicates the strain has undergone evolutionary changes that favored survival of these host cells and increased viral replication in DCs which are found in key organs, including the testis.

Contrary to Dengue virus, ZIKV immunopathology is not based on the release of overwhelming amounts of cytokines and chemokines [88]. As reported for other flaviviruses [89], minimal up-regulation of MHC-II, co-stimulatory molecules and pro-inflammatory cytokines secretion was detected in ZIKV-infected DCs in vitro and ex vivo. So it appears that ZIKV impairs the ability of DCs to present antigens and to prime T-cell responses, interfering in the crosstalk between innate and adaptive immune responses [87]. Similar results were obtained with historical strains of ZIKV in this study in a clear demonstration of a preserved mechanism of viral interference with host innate immune responses.

Infected human monocyte derived DCs produce minimal amounts of IFNα and IFNλ1 and fail to produce IFNβ, despite increased transcription of their respective genes [87]. Plasmacytoid DCs also produce low amounts of IFNα after ZIKV infection. ZIKV infection seems to compromise the ability of human DCs to translate IFN type I and III mRNAs into proteins [87], but the production of other proteins involved in viral RNA recognition (RIG-1, MDA5 and LGP2) and antiviral effectors proteins (IFIT1, IFIT3 and viperin) are up regulated meaning that interferon independent antiviral responses persist as reported for West Nile virus [90] and might be key to control of human infections by ZIKV.

In the case of IFNs, it was found that Zika virus antagonizes the phosphorylation of STAT1 and STAT2 which are transcription factors downstream the IFN type I signaling pathway, limiting even further the role of these IFNs in the control of viral replication in DCs [87]. It has also been shown that ZIKV NS5 targets STAT2 for proteasomal degradation in humans [91]. ZIKV ability to escape interferon mediated antiviral responses in DCs might result in greater viral replication, higher viral load, and enhanced ability of the virus to reach the BTB to be sexually transmitted, especially after infected macrophages act to make this barrier leakier.

#### 3.1.3. Zika Virus Interactions in the Peritubular Space and the Seminiferous Tubule Wall

Zika virus AXL co-receptor has been identified by immunohistochemistry in human peritubular myoid cells (Figure 2) opening up the possibility that after replicating in cells present in the interstitial space, such as DCs and macrophages, ZIKV could adsorb and infect these peritubular cells before reaching germ cells and the Sertoli cell layer [92]. A testicular germ cell line (833KE) is permissive to ZIKV infection by contemporary and historical strains, but viral load is higher with the historical strain [93]. No cytopathic effects were noted. Nonetheless, only a small percentage of germ cells was infected and low viral NS1 expression was observed in both cases (one and five percent, respectively). These results point to a progressive evolution of ZIKV strains to cause less damage to human germ cells allowing for persistent spermatogenesis and shedding of viral particles by infected individuals.

Using primary human Sertoli cell (Figure 2) culture and a Sertoli Cell in vitro barrier model, Siemann and co-workers [84] demonstrated that Sertoli cells are highly susceptible and permissive to ZIKV. Replication was detected after 24 h and was still high nine days post-inoculation when the cells started to die in culture. In the human body, under normal physiological conditions, the infection might persist longer favoring the infection of spermatogonia, spermatids and spermatozoa and ultimately lead to shedding of ZIKV in semen and sexual transmission. Interestingly, the same results were seen when Sertoli cells were inoculated with West Nile virus. This is not the case for all flaviviruses, as the replication of Dengue virus in these cells was always lower. None of these viruses caused any cytopathic effect or Sertoli cell death indicating that ZIKV will probably reach the lumen of the seminiferous tubule through budding from the apical membranes of these cells at the end of the replication cycle.

The infection of Sertoli cells induced the production of only modest amounts of IFN-α—a cytokine involved in the activation of the antiviral state. ZIKV evades this antiviral mechanism using viral NS5 to induce the proteasomal degradation of STAT2 in humans (but not in mice) inhibiting the activation of hundreds of interferon-regulated genes, in a ubiquitination independent fashion [91]. This evasion of a potent innate antiviral response could constitute a ZIKV strategy to increase intracellular replication in neighboring Sertoli cells and to build up a viral reservoir in the Sertoli cell layer in the MRT.

Infected Sertoli cells upregulated the production of multiple pro-inflammatory cytokines such as IFN-γ, IL-1α e β, IL-6, IL-8 and TNF-α. Chemokines, used to attract leukocytes to sites of infection, were also produced by ZIKV-infected Sertoli cells and RANTES (CCL5), fractalkine (CX3CL1), IP-10 (CXCL10) and GRO (growth related oncogene CXCL1) were found in the supernatant after 46 h of inoculation [84]. Thus, Sertoli cells can activate a strong innate immune response against ZIKV under laboratory conditions and probably represent a major source of cytokines in infected males. It has been shown that TNF-α increases the production of IP-10 in mouse testis and that this chemokine induced germ cell apoptosis in vivo in Mumps virus-infected testicles [94]. If a similar mechanism occurs in humans, ZIKV infection could have negative impacts on male fertility altering specific stages of the seminiferous epithelial cycle leading to a decline in total sperm count as has been already reported in long term shedders of this virus in semen [55].

The infection of Sertoli cells by ZIKV also induced the expression of the adhesion molecules VCAM-1 and ICAM-1 [84], known to be important for leukocyte migration across the endothelium, the blood–brain barrier, the blood–epididymal barrier and the BTB. However, ZIKV infection did not alter the expression of tight junction proteins or that of metalloproteinases, which would facilitate leukocyte and viral trafficking towards the lumen of the seminiferous tubule. These junctions are under strict control during spermatogenesis to allow the migration of the spermatocytes in the seminiferous tubule wall [95]. Even though ZIKV does not directly alter Sertoli cell’s tight junctions, free ZIKV particles can transmigrate an in vitro SC barrier model and reach the adluminal side much more efficiently than an in vitro blood–brain barrier. Thus, infected Sertoli cells might serve as ZIKV reservoirs in vivo contributing to viral persistence in the MRT, constantly replenishing the semen with new viral particles and increasing the opportunities for sexual transmission.

Interestingly, the exposure of Sertoli cells to pro-inflammatory mediators produced by ZIKV-infected macrophages led to the degradation of ZO-1, one of the proteins found in tight junctions, with the resultant increase in permeability [84]. These results illustrate perfectly well the concept that the SC barrier integrity and function are under the control of a complex interplay between cytokines and other immune factors released in the testis during infectious processes.

A cohort study conducted in Puerto Rico detected that men in the acute phase of ZIKV infection have an increased level of Follicle Stimulating Hormone (FSH) and low levels of Inhibin β. This is a strong evidence that the infection damages Sertoli cells lowering their production of Inhibin β. There is a good correlation between low levels of Inhibin β, the arrest of spermatogenesis and testicular damage [96]. In fact, total sperm count, and total motile sperm count decreased while sperm abnormalities increased in these individuals. Inhibin β can become undetectable when germ cells are eliminated, but that would not be a good strategy for ZIKV which depends on host cell survival for sustained replication [97] and sexual transmission. Testosterone levels were not significantly altered by the infection a finding that corroborates the absence of reports of ZIV infection in human Leydig cells [55]. Importantly, these alterations in sperm counts and morphology tended to subside with minor alterations persisting until the end of the study period (120 days) revealing that fertility will probably be preserved in males who have been infected by ZIKV.

ZIKV antigens have been detected by immunostaining in the head of mature human spermatozoa [98]. These antigens found in spermatozoa might have originated from the infection of germ cells, spermatogonias, spermatids or spermatozoa. We will discuss this latter in this article when we consider the detection of ZIKV in semen.

### 3.2. Zika Virus in the Epididymis

The human epididymal epithelial lining (Figure 2) expresses the AXL co-receptor for ZIKV [92] and this could result in the infection of these cells by free ZIKV particles arriving from the seminiferous tubules, as has been already detected in mouse models [99]. Viral replication in the epididymal epithelium would amplify the infection in the MRT and could be a plausible explanation for the high viral loads detected in semen. In one study, the viral load in semen was 100,000 times higher (8.6 × 10^10^ copies/mL) than that in whole blood [28].

As described above in the testis, the virus could also reach the epididymis from the blood and infected interstitial cells such as macrophages and DCs and gain access to the spermatozoa in the epididymal lumen. Here, the virus could adsorb to the membranes of spermatozoa or infect these cells in the acute phase of the disease when spermatozoa traffic for approximately 12 days in the lumen of the epididymis [55].

The DC population in the normal epididymal intertubular space is primarily composed of immature plasmacytoid and myeloid DCs [100]. These cells change to mature phenotypes upon persistent inflammation of the epididymis and could affect the integrity and permeability of the fragile BEB (Blood–Epididymis Barrier) promoting a Th17 inflammatory response during the course of ZIKV infection, as has been demonstrated for other infectious agents [101]. As a result of inflammation, DCs could meet self-antigens present on spermatozoa, capture and present them to auto-reactive T cells increasing the chances for fertility disorders. This hypothesis has yet to be tested in long term follow up studies of ZIKV-infected males. The development of new methods with higher sensitivity to detect ZIKV RNA in the epididymis of mouse will probably contribute to further studies of the interactions of ZIKV in this organ in humans [102].

### 3.3. Zika Virus in the Prostate Gland and in the Seminal Vesicle

Inflammation and infection of the testis and epididymis seem to be more relevant for male reproduction than inflammation or infection of accessory glands [83]. However, in non-human primates ZIKV persists in the prostate and seminal vesicle after viremia has been resolved [103]. In mouse models, ZIKV does not infect the prostate and seminal vesicle [104], possibly because AXL or other flavivirus receptors are not present on the surface of the cells in these two glands.

Evidence that these anatomical sites might be permissive to ZIKV comes from two studies of ZIKV shedding in vasectomized men. In these cases, the surgical procedure cuts and seals the ductus deferens that carries spermatozoa from the testis and epididymis towards these glands. Therefore, the source of the virus would have to be ahead of the surgical incision in the prostate, seminal vesicles or bulbourethral glands. One cannot rule out a possible role of urethral epithelium in ZIKV replication in the MRT.

In the first case report, infectious ZIKV particles were detected in the ejaculate of a vasectomized man, 69 days after symptom onset with probable transmission to the sexual partner [105]. In the second case report, ZIKV RNA was detected in the ejaculate of a vasectomized man, up to 77 days after symptom onset and the virus was proven to be infectious in cell culture up to 21 days after symptom onset [60]. No abnormal findings could be noted on digital palpation of the prostate of the vasectomized men and the level of the Prostate Specific Antigen (PSA) was normal suggesting that ZIKV infection, if present in the prostate of this patient, did not significantly alter the inflammatory status or the physiology of the gland. The microscopical analyses of the ejaculate revealed the absence of spermatocytes indicating that the surgical procedure had been successful. Round cells, most probably leukocytes, were detected. Some of these round cells were peroxidase positive (0.05 × 10^6^/mL) suggestive of neutrophil infiltration which has also been described in *Rhesus* macaques infected by ZIKV [106]. These round cells could be harboring ZIKV particles attached to their surface or carrying the virus inside their cytoplasm, but this was not determined in this study.

It is important to highlight that prostatitis—a disease commonly associated with bacterial infections—has been reported in male patients infected with ZIKV and DENV [107,108,109,110] and that these viral agents must be remembered in the differential diagnosis of this disease in endemic areas and in travelers returning from countries where these viruses circulate. This symptom will prompt the patient to seek medical assistance creating an opportunity for sampling for ZIKV detection. Hematospermia, microhematospermia [111] and perineal pain are also indicative of prostatitis and have been reported in males with ZIKV infection [27,112,113]. However, most male infections course without this complaint [27,31,41,42,114] suggesting that only a small number of male patients will probably have ZIKV in the prostate or that specific host–virus interactions might be involved in the control of the infection in the prostate. It would be advisable in such cases to needle biopsy the prostate to determine the inflammatory status of the gland and to test for the presence of Zika virus antigens.

A metastatic prostatic adenocarcinoma cell line (LNCaP) proved to be permissive to ZIKV infection [93] with greater expression of the viral NS1 protein than seen in a testicular cell line (833KE). Viral load and the number of infected cells significantly increased from day 1 to day 5 after inoculation. No cytopathic effects were noticed after infection of these cells with a contemporary or historical ZIKV strain pointing to host cell survival and persistent infection potential of these cells. Altogether, these results suggest that the prostate might be a more efficient site for ZIKV amplification and persistence than initially thought. Additional studies are needed to determine the precise potential of this gland to serve as a viral reservoir in infected male patients.

### 3.4. Zika Virus in Semen

Semen is the vehicle that carries ZIKV particles from the MRT to the FRT. Viruses can travel as free particles in seminal plasma, adsorbed to cell membranes or inside the cytoplasm of leukocytes and spermatozoa. Many case reports of sexual transmission of ZIKV from male-to-female and from male-to-male have been published in a clear demonstration of the infectivity of ZIKV particles present in human semen [17,26,33].

Shedding of ZIKV particles in semen is variable between individuals [98,115]. In a prospective cohort in Puerto Rico, shedding was detected in 73% of symptomatic men and started as early as seven days post-infection [55]. Shedding can occur concomitantly with virus presence in urine and blood or after viral clearance for these fluids [22,115]. Viral shedding can also be intermittent or persistent [114] for up to 188 days (six months) with viral load reaching 50,000 copies/mL in the first two weeks and ranging from 1000 to 10,000 copies/mL at the end of this period [54]. The final shedding date indicates elimination of the virus by the immune system [115] but might as well represent one more episode of intermittent shedding.

One interesting aspect of ZIKV shedding in semen is that asymptomatic patients can sometimes excrete longer (68 days) than symptomatic ones [113]. For that reason, men diagnosed with ZIKV infection should avoid attempting conception for at least six months and keep systematic use of condoms in all sexual intercourses in this period. The high rate of asymptomatic infection following ZIKV infection, estimated in 80% of infected individuals [116], leads to a gross underestimation of the risk for sexual transmission.

ZIKV RNA can be detected in the seminal plasma (the liquid supernatant after centrifugation of semen) and in the cellular components of semen. Interestingly, in all negative semen samples no RNA could be detected in the cellular pellets. Nonetheless, viral RNA was detected in two percent of seminal plasma negative samples. Furthermore, in 16% of the seminal plasma positive samples, no RNA could be detected in the cells [55]. These results confirm that ZIKV can travel free in seminal plasma or attached/inside the cells present in semen. Similar results were reported elsewhere [117]. Studies must be conducted to determine if free virus particles can cause infection in the FRT or if the virus must be transmitted inside spermatozoa during fertilization.

Spermatozoa were purified by gradient centrifugation and ZIKV RNA was found in 25% of the samples, all of which came from shedders with high viral loads (>5 log copies per mL) in seminal plasma suggesting that higher viral loads lead to higher infection rates in spermatozoa [55]. Moreover, 64% of the motile spermatozoa samples tested positive for ZIKV RNA and the virus from one of the samples could be cultured in Vero E6 cells. Other researchers detected viral RNA only in the cellular fraction of semen and were unable to culture the virus [54]. These results clearly demonstrate that spermatozoa from infected males maintain their ability to swim and can carry infectious virus particles to the FRT during fertilization which could lead to ZIKV congenital syndrome in a percentage of the infected fetuses [118]. Unfortunately, one cannot be sure of the precise localization of ZIKV particles in this study, either adsorbed to or inside spermatozoa.

The presence of ZIKV antigens inside the head of spermatozoa was unambiguously demonstrated using immunohistochemical techniques associated with confocal and emission depletion microscopy [98]. At least 3.52% of the spermatozoa carried the virus and the authors argued that the virus might have originated from Sertoli cells which are known to have the AXL co-receptor. However, in mice AXL is not essential for ZIKV infection, clinical signs, viral load, tropism or histological changes in major organs [119]. Thus, the virus could have infected germ cells, spermatocytes or spermatozoa in the testis or during its traffic and storage in the epididymis as hypothesized earlier in this article.

Much more concern arises when one considers the presence of infective ZIKV particles in spermatozoa [55] because they can directly deliver the virus into the cytoplasm of the oocyte during fertilization [120] which can lead to miscarriage or congenital ZIKV syndrome. Intravaginal exposure to ZIKV in mouse models can lead to fetal brain infection [121]. There is a possibility that during outbreaks, such as the one in French Polynesia and the more recent one in the Americas, this mode of transmission was responsible for some of the microcephaly cases reported. In Brazil, where the use of condom is not universal, almost 3000 cases of congenital ZIKV syndrome have been reported and confirmed by the Ministry of Health [122]. With increased knowledge and information, men could plan to avoid unprotected sexual intercourses, semen donation or planned pregnancy for at least six months after disease onset.

Some negative effects of Zika virus presence in the MRT have been reported [55]. Shedders had significantly lower semen volume, lower total sperm count and lower total motile sperm at least in one occasion and a significant increase in spermatozoa anomalies in two occasions. One can only infer that the infection of the MRT interferes, at least temporarily, with the level of spermatogenesis, sperm morphology, sperm fitness for fertilization and affects the production of seminal plasma by the annexed glands. Further studies are urgently needed to elucidate the long-term consequences of this infection in human males.

In terms of ZIKV epidemiology, shedding of infective particles in semen is what matters the most not only because of its possible adverse effects on the fetus, but also because it allows the transmission of the virus in areas where the mosquito vectors do not thrive. Inoculation of virus particles from semen samples in cell cultures in vitro and checking for increasing viral load has been used to determine viral viability. Using this approach, it was demonstrated that infective particles were shed up to 69 days after symptom onset [105,115] and sexual transmission was probable in one of these cases [105]. Sexual transmission has been reported to occur as late as 41 days after the onset of illness in an index case [23].

The detection of infectious virus particles has not always been successful and might be due to technical issues. Negative cultures might reflect the absence of infective particles, technical inadequacy, insufficient technical skills, viral neutralization, viral persistence inside semen cells, viral destruction by processing, among other possibilities. A good approach is to free all possible ZIKV particles present in the cell fraction before inoculation onto the cell layer in vials.

It is known that the semen can influence the vaginal environment, the female immune responses, the establishment of a successful pregnancy and viral transmission [123]. Since infected cells in the MRT produce several cytokines and chemokines it would be interesting to measure their concentrations in semen samples of ZIKV-infected males to determine their potential effects on the FRT and pregnancy outcomes.

## 4. Conclusions

ZIKV, a sexually transmitted virus, invades the male reproductive tract and interacts with immune cells, germ cells and spermatogonia being detected inside motile spermatozoa and posing a serious threat to human reproduction. Long term shedding of high loads of infectious viral particles in semen suggests that this virus can persist in the male reproductive tract. Detailed studies are needed to identify the location of host cells permissive for ZIKV replication in the male reproductive tract, the viral reservoirs and the extent of viral shedding and infectivity in the male population. Further research is also needed to determine if the virus infects and replicates in annexed glands and in the urethral epithelium. Some of the immune responses elicited by ZIKV in the male reproductive tract have been described, but there is much more still to be discovered about viral immune evasion and persistence in this anatomical site to pave the road for appropriate vaccine and antiviral development.

## Figures and Tables

**Table 1:** Important advances in the history of Zika virus (ZIKV) sexual transmission.

Date	Advances	Reference
May 2011	First report of ZIKV probable sexual transmission	[16]
May 2014	Two cases of probable sexual transmission reported	[26]
February 2015	Infectious ZIKV particles detected in semen samples	[27]
March 2016	High viral load reported in semen sample	[28]
May 2016	Zika virus presence in semen confirmed	[29]
April 2016	Zika RNA found in semen of asymptomatic blood donors	[30]
April 2016	First report of male-to-male sexual transmission	[31]
July 2016	First report of Zika RNA in cervical mucus	[32]
July 2016	First report of suspected female-to-male sexual transmission	[33]
